# Solidification-Induced Wear Behavior of Composite Coatings Fabricated via Laser Cladding

**DOI:** 10.3390/ma18112521

**Published:** 2025-05-27

**Authors:** Zidan Wang, Xue Yan, Zhiqiang Liu, Youwei Wang, Kailun Zheng, Qian Bai

**Affiliations:** State Key Laboratory of High-Performance Precision Manufacturing, Dalian University of Technology, Dalian 116024, China; wangzidan21@mail.dlut.edu.cn (Z.W.); yanxue@mail.dlut.edu.cn (X.Y.); 1512618934@mail.dlut.edu.cn (Z.L.); wangyouwei@mail.dlut.edu.cn (Y.W.); zhengkailun@dlut.edu.cn (K.Z.)

**Keywords:** WC-enhanced iron-based composite coating, laser cladding, solidification behavior, toughness, overall performance

## Abstract

The Fe-based composite coating reinforced with WC has broad application in critical components of the steel metallurgy industry. However, the effect of WC content on the microstructure and, thus, comprehensive mechanical performance of these coatings remains insufficiently understood. In this study, laser cladding was used to fabricate Fe-based coatings with varying WC contents to systematically investigate their influence on solidification behavior, microstructural evolution, and wear performance. A novel evaluation method integrating mechanical properties and wear resistance was proposed to assess overall performance. The results reveal that increasing WC content leads to improved hardness and wear resistance, with reduced toughness. At 40 wt.% WC, the coating exhibited approximately 40% higher hardness and 27% better wear resistance compared to the 20 wt.% WC coating, achieving a better balance between strength and ductility. This study offers new insights for optimizing wear-resistant coatings in steel metallurgy industrial applications.

## 1. Introduction

The extrusion mandrel plays a crucial role in the manufacturing process of steel metallurgy products. During the extrusion process, the mandrel is in direct and sustained contact with high-temperature billets, enduring the effects of high-intensity alternating stress and cyclic thermal loads. These harsh conditions can easily cause fracturing of the extrusion mandrel [1]. To guarantee the integrity of manufacturing and the efficiency of the production process, the mandrel must exhibit superior high-temperature wear resistance and impact toughness. Surface coating technology stands out as an efficient strategy for enhancing the performance of extrusion mandrels, with composite coating techniques offering a tailored blend of diverse material attributes [2]. The predominantly used alloy powders, such as nickel-based, cobalt-based, and iron-based, boast impressive tensile strength and corrosion resistance, making them the preferred choice for matrix powders [3,4]. Ceramic particles, with their exceptional hardness and affinity for metallic melts, are considered an ideal hard particle additive material [5,6]. Ceramic particle-reinforced composite coatings combine the exceptional toughness of the matrix material with the inherent hardness and wear durability of ceramic particles, profoundly boosting the resistance to wear and high-temperature resilience of the coating [7].

Enhancing the volumetric concentration of the ceramic reinforcement phase boosts the performance of composite coatings. Yang et al. [8] employed high-speed laser cladding technology to fabricate Fe/Ti_3_SiC_2_ coatings. The findings indicated that the interface between the coating and the matrix primarily consisted of columnar, dendritic, and planar crystals. Compared to the matrix, the wear loss exhibited by the composite coating underwent a substantial reduction of 94%. Meng et al. [9] utilized the Electrothermal Heating-Laser Induced Hot Coating (EH-LIHC) technique to fabricate composite Ni60-WC coatings with various WC concentrations. The findings indicated that the Ni35-WC65 coating emerged as the one with the greatest abundance of WC particles and surface carbides, leading to a notable enhancement in its damage tolerance and resistance to wear. Zhao et al. [10] prepared WC-phase-reinforced alloy coatings. The findings indicated that as the WC content rose, the carbide phase morphology shifted from being discrete to coarse. The coating with 40% WC content displayed robust wear resistance at room temperature, 400 °C, and 500 °C.

However, these analyses of coating performance predominantly center on individual wear performance. Metallurgical critical components like extrusion mandrels are subjected to intricate conditions involving repeated friction and impact loads, necessitating both wear resistance and adequate strength and toughness. Meanwhile, the influence of the distinct solidification behavior of the coating on its performance remains largely unexplored. Therefore, investigating the effects of various solidification behaviors on coating performance and developing overall performance evaluation criteria for the coating is crucial for enhancing its overall performance. Additionally, their moderate melting point and flowability facilitate the formation of uniform and dense coating structures [11]. WC ceramic particles are ideal as hard phase additives owing to their elevated melting point, low thermal expansion coefficient, and exceptional hardness [12,13].

In this study, laser cladding technology was used to fabricate WC-enhanced iron-based composite coating (WC-M2) with WC mass fractions of 20 wt.%, 30 wt.%, 40 wt.%, and 50 wt.% on H13 steel. The study analyzed the microstructure, mechanical properties, and tribological behavior of the coatings, explored the impact of different solidification conditions on wear performance, and proposed an evaluation method for the overall performance of composite coatings. These results may serve as a guide for optimizing the performance of the coating.

## 2. Material and Experimental Procedures

WC ceramic particles and M2 iron-based alloy powder were utilized in the fabrication of WC-enhanced iron-based composite coating via laser cladding technology. The chemical composition of M2 and H13 is shown in Table 1. Mixed powders with varying WC contents of 20%, 30%, 40%, and 50% by weight were prepared and labelled as WC20, WC30, WC40, and WC50, respectively. The powder mixture was blended using a planetary ball mill (QM-3SP4, Nanjing Nan Da Instrument, Nanjing, China), with ZrO_2_ balls for grinding, a ball-to-powder weight ratio of 1:1, and a grinding speed of 200 rpm. The coating procedure was implemented employing an AFS-D600 machine (from Long yuan AFS Co, Ltd., Guangzhou, China). Continuous wave laser technology was applied in the laser cladding process to deposit WC-M2 coatings onto H13 steel substrates. The WC particles and M2 alloy powders both have a particle size distribution of 40–45 μm. The specific experimental parameters are detailed in Table 2. In view of the high melting point and significant proportion of WC in the coating, a combination of high laser power (2000 W), slow scanning speed (5 mm/s), and large spot diameter (2 mm) was adopted to provide adequate energy input and promote uniform, high-quality coating formation.

Samples for microstructural analysis were embedded in phenolic resin, and their surfaces were polished to a consistent texture using SiC sandpaper ranging from #180 to #2000. To analyze the microstructure, a Scanning Electron Microscope (SEM, SU5000, Hitachi High Technologies, Tokyo, Japan) equipped with an Energy-Dispersive Spectrometer (EDS, Ultim Max, Oxford Instruments, Abingdon, UK was employed). In addition, ImageJ software (version 1.5) was used to analyze SEM images to determine the count, average size, and volume percentage of WC particles. An EBSD (Electron Backscatter Diffraction, SUPRA^TM^55, Carl Zeiss, Oberkochen, Germany) test was conducted to collect quantitative crystallographic information of the samples. Transmission Electron Microscopy (TEM) observations were conducted on the WC40 specimen using a Tecnai G2 F20 instrument (FEI, Hillsbore, OR, USA) to investigate the fine-scale microstructural features of WC particles. X-ray diffraction (XRD) measurements were conducted using a D8 Advance X-ray diffractometer (Bruker AXS, Karisruhe, Germany) with monochromatic Cu-Kα radiation at a wavelength of 1.54056 Å, scanning from 30° to 90°. HighScore Plus software (version 3.0.5) was employed for phase identification using the standard Powder Diffraction File (PDF) database provided by the International Centre for Diffraction Data (ICDD, Newtown Square, PA, USA). Microhardness tests were conducted with an MVS-1000Z Vickers hardness tester (Shanghai Jingjing Instrument Co., Ltd., Shanghai, China), applying a 0.5 kg load for 15 s per test; five points were sampled horizontally across each region. To evaluate the plastic behavior of the coating, we employed the NHT^3^ nano-indentation(Anton Paar, Graz, Austria) tester (step500) to conduct micro-indentation tests on the coating surface, utilizing a spherical indenter to consistently apply a 500 mN force. Nano-indentation tests were repeated on three parallel samples for each WC content, and five indentations were performed on each sample to ensure statistical reliability. To assess the tribological performance of the coating, we conducted a high-temperature friction test on its surface utilizing the UMT Tribolab friction tester (Bruker, MA, USA), with a counter body of a 9.5 mm diameter high-precision Si_3_N_4_ ceramic ball. The specific wear test parameters are detailed in Table 3. The 3D topography of the wear tracks was inspected using a three-dimensional surface optical profiler (NewView9000, Zygo Corporation, Middlefield, CT, USA). Each WC content group was tested on three parallel samples to ensure data reliability.

## 3. Results and Discussion

### 3.1. Macroscopic Characteristics

**Figure 1:**
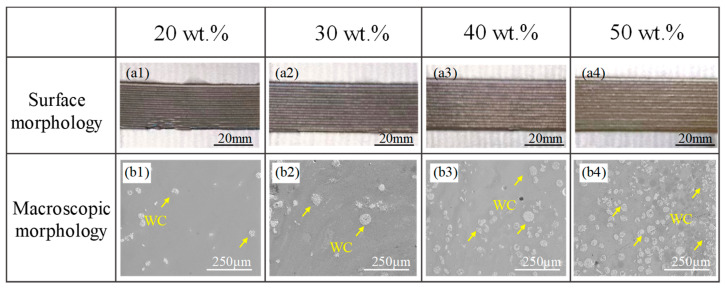
The surface morphology of the coatings: (**a1**) WC20; (**a2**) WC30; (**a3**) WC40; (**a4**) WC50; the macroscopic morphology of the coatings: (**b1**) WC20; (**b2**) WC30; (**b3**) WC40; (**b4**) WC50.

Figure 1(a1–a4) display the surface morphologies of the WC-M2 composite coatings. The coating surfaces are even and well-shaped and are devoid of any visible cracks or pores. The variation in macroscopic morphology with increasing WC content is depicted in Figure 1(b1–b4). It is evident that the coatings feature white spherical particles, and with the rise in WC content, the quantity of these particles progressively increases, leading to clustering.

**Figure 2:**
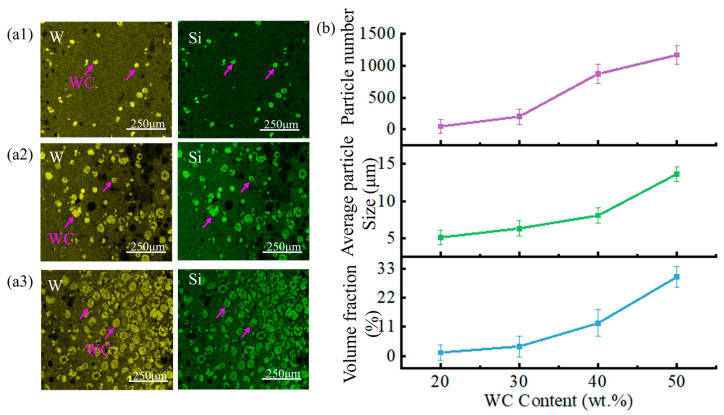
Element distribution of the coatings: (**a1**) WC20; (**a2**) WC40; (**a3**) WC50; (**b**) number of particles per unit area, average particle size, and volume fraction of the coating.

**Figure 3:**
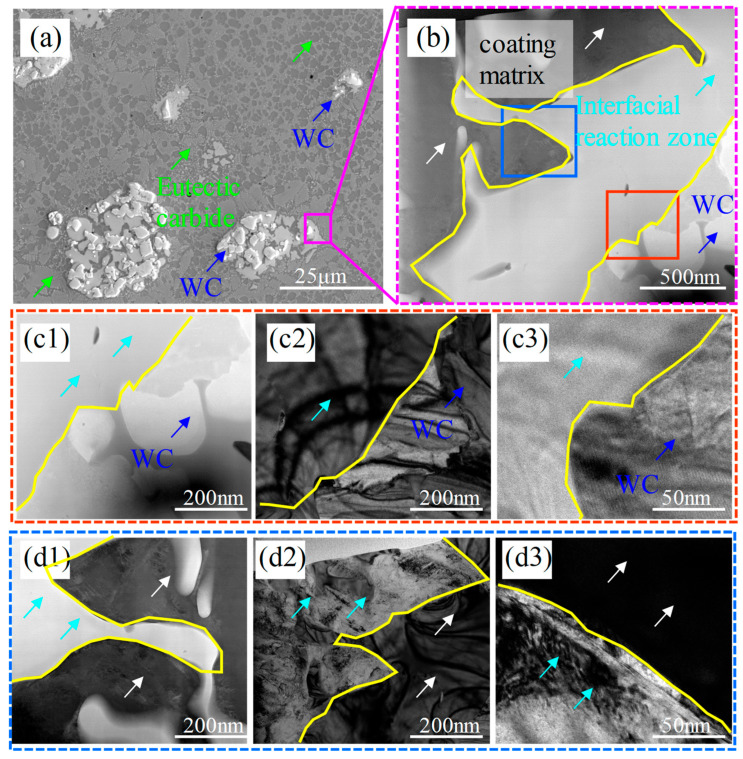
(**a**) Morphology of WC particles within the coating of the WC40 sample; (**b**) enlarged morphology of the WC particle edges; WC particles and interface reaction zone: (**c1**) TEM morphology; (**c2**) TEM dark-field morphology; (**c3**) HRTEM morphology; interface reaction zone and coating matrix: (**d1**) TEM morphology; (**d2**) TEM dark-field morphology; (**d3**) HRTEM morphology.

The EDS results are presented in Figure 2(a1–a3), where the particles exhibit higher concentrations of W and Si elements. Due to the limitations of EDS in detecting light elements such as carbon, the carbon in WC was not detected. In contrast, silicon was found in the EDS, which is attributed to the fact that the silicon element was one of the original compositions in the M2 matrix and was diffused into the WC particles at high temperatures, as shown in Figure 3b. Image J software was used to analyze SEM images and determine the count, average size, and volume percentage of WC particles. At least five areas were measured per sample, and the averages were tabulated as the results, as depicted in Figure 2b. As the WC content rises, there is a corresponding increase in the count, diameter, and volumetric proportion of the WC particles; this elevated WC particle density impedes the heat dissipation routes [14]. The complexity of the heat conduction process restricts the effective transfer of heat, leading to some WC particles not fully absorbing heat to achieve complete decomposition, thereby increasing the quantity of undecomposed WC within the coating. Moreover, the limitation of heat conduction can induce substantial changes in temperature gradients and cooling rates within the coating [15,16], making irregularly shaped particles more prone to remaining undecomposed, thus increasing the average size of the undecomposed particles. At 40 wt.% WC content, the arrangement of WC particles throughout the coating is consistent, as depicted in Figure 2(a2); at 50 wt.%, WC particle aggregation is observed in the coating, as depicted in Figure 2(a3).

### 3.2. Microstructural Characteristics

#### 3.2.1. Grain and Eutectic Carbide

Since a more uniform microstructure and an optimal balance between hardness and toughness can be achieved by using WC40 [17], TEM analysis on the WC40 specimen was performed. A pronounced fragmentation of the WC particles is apparent in Figure 3a. A detailed magnification was conducted at the boundary where the WC particles react with the M2 matrix, as illustrated in Figure 3b. The image distinctly reveals a demarcation zone between the WC particles and the M2 matrix, marking it as the interface reaction zone. During this process, elements such as Si from the M2 matrix diffuse into the WC particles, as evidenced in Figure 2(a1–a3). Figure 3(c1–c3) depict the TEM images, along with HRTEM (High-Resolution Transmission Electron Microscopy) images, of the WC particles and the interface reaction zone, while Figure 3(d1–d3) present similar imagery for the interface reaction zone and the coating matrix. The TEM images reveal a clear demarcation of light and dark boundaries between the WC particles, the interface reaction zone, and the coating matrix, indicating significant phase and compositional changes. This suggests that the reaction layer is not the original WC particles but rather a new reaction product formed around the WC particles due to their erosion by Fe elements and the subsequent chemical reaction.

Figure 3a also reveals that the eutectic carbides in proximity to the particles are more concentrated than those in the coating matrix. When exposed to high-energy laser beams and the extreme temperatures of molten metal, the WC particles break down, liberating elements like W and Si. W and Si atoms along the particle edges react with the Fe-based melt to create the interface reaction zone. Meanwhile, a fraction of these elements dissolves into the Fe-based melt, resulting in the precipitation of eutectic carbides. Surrounding the decomposed WC particles, the elevated concentrations of these elements result in an increased formation of carbides.

**Figure 4:**
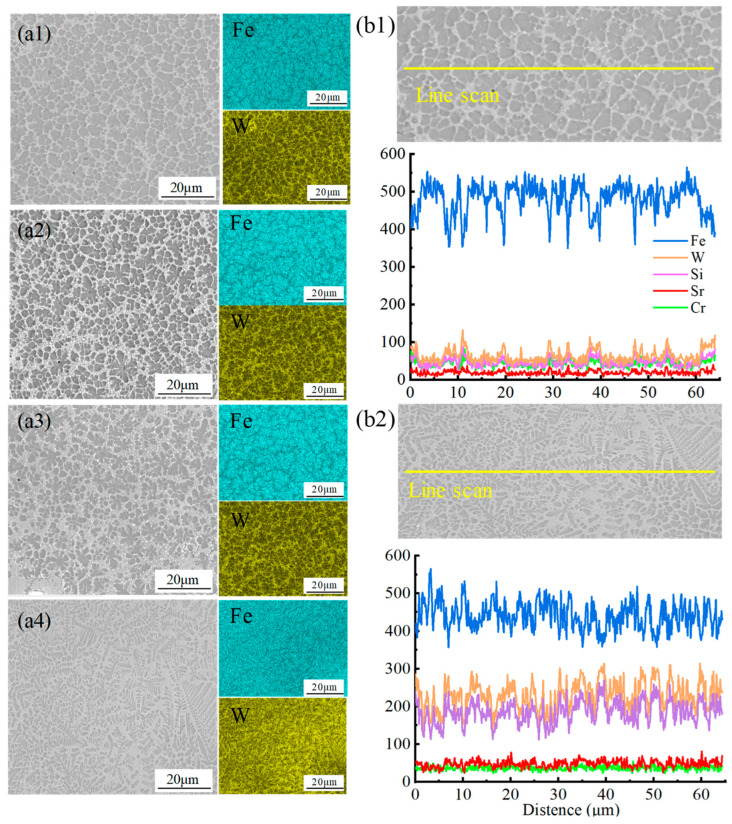
Microstructure morphology and arrangement of Fe and W elements in the coatings: (**a1**) WC20; (**a2**) WC30; (**a3**) WC40; (**a4**) WC50; EDS line scan results of the coatings: (**b1**) WC20; (**b2**) WC50.

The line scan analysis of the microstructure of the coating is presented in Figure 4(b1,b2), revealing a significant contrast in the concentration of W and Si elements between the samples WC50 and WC20, with WC50 exhibiting a higher concentration. The coating microstructure displays two distinct solidification behaviors. When the WC content ranges from 20 wt.% to 30 wt.%, as depicted in Figure 4(a1,a2), the coating microstructure demonstrates the first type of solidification behavior, characterized by a fine network of eutectic carbide structures. EDS spectral analysis indicates an enrichment of W within the network carbides, while the grains are abundant in Fe. At a reduced WC content within the powder, numerous WC particles damaged by heat break into smaller fragments, decompose within the melt pool, and produce elements like W and C. Fewer WC particles are retained, and the atoms in the melt pool randomly initiate nucleation, forming network eutectic carbides [18].

When the WC content ranges from 40 wt.% to 50 wt.%, as shown in Figure 4(a3,a4), the coating microstructure exhibits the second type of solidification behavior. Where the W element content significantly increases, and the eutectic carbides transition from fine network structures to blocky or floral structures. As WC content rises, the amount of W and C elements in the molten pool surges, leading to a higher retention of WC particles. These particles act as nucleation sites for the molten material, promoting the likelihood of atom nucleation on the WC particle surfaces [19]. As the solidification process advances, the grains that nucleate around the WC particles grow and interlock, ultimately forming an eutectic structure primarily composed of W, Fe, and C [20]. Concurrently, with the augmentation of eutectic carbide content, the grains in the polyhedral eutectic become finer.

#### 3.2.2. Phase Composition

**Figure 5:**
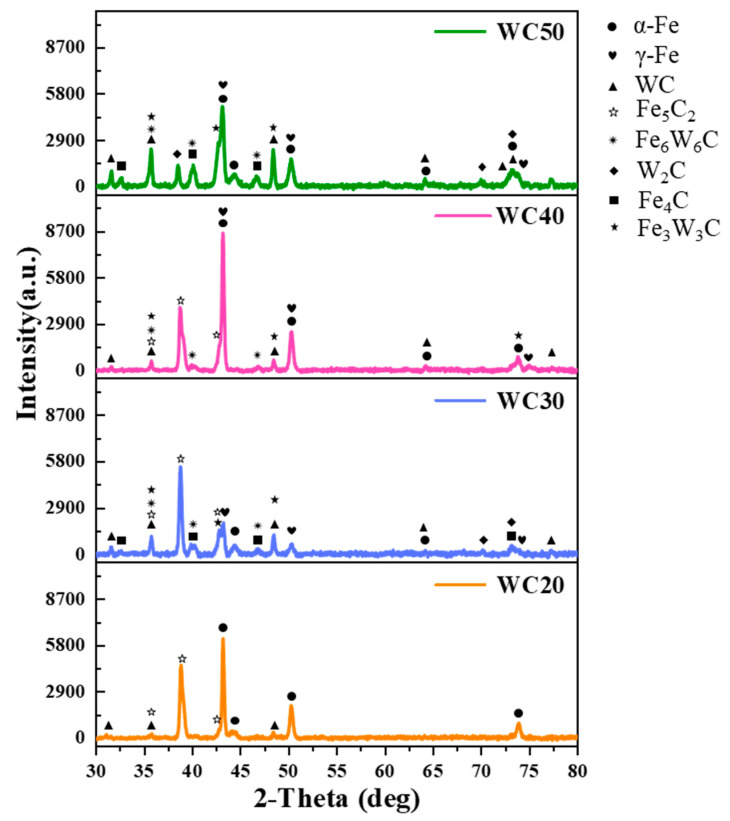
XRD analysis of WC-M2 composite coatings.

The XRD analysis for the WC-M2 composite coating, as depicted in Figure 5, reveals that α-Fe and γ-Fe constitute the predominant matrix phases within the coating. Additionally, the iron-based solid solution plays a pivotal role as both a binder and a substrate for the hard carbide phase that is present [21]. As the WC content increases, the concentrations of W and C elements within the molten pool intensify, fostering more profound interactions with the Fe-based liquid. Consequently, the formation of diverse carbide phases is promoted, such as MC (where M represents metallic elements like Fe, Mn, etc.) and Fe-W-C phases, resulting in an increase in the diversity and intensity of carbide diffraction peaks.

In the XRD patterns, some diffraction peaks are labeled with multiple phase symbols. This is because different phases may share similar lattice spacings, resulting in the superposition of reflections at the same 2θ positions. Therefore, the superposed peaks were assigned based on the matched PDF cards and verified through HighScore Plus analysis. This method is commonly adopted in XRD phase identification.

**Figure 6:**
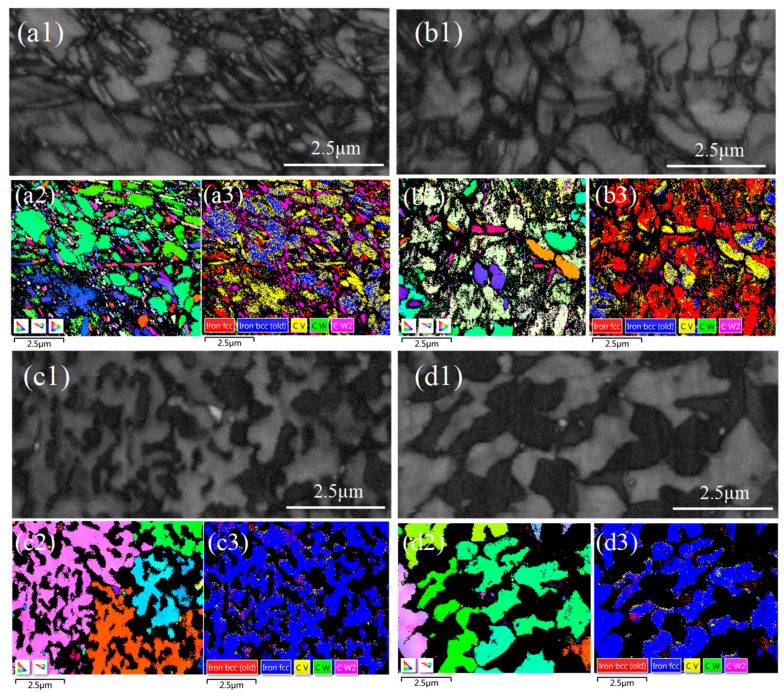
Backscatter diagram of the WC-M2 composite coating: (**a1**) WC20; (**b1**) WC30; (**c1**) WC40; (**d1**) WC50; IPF X map: (**a2**) WC20; (**b2**) WC30; (**c2**) WC40; (**d2**) WC50; phase map: (**a3**) WC20; (**b3**) WC30; (**c3**) WC40; (**d3**) WC50.

The EBSD results are illustrated in Figure 6. Figure 6(a1,b1,c1,d1) depict the backscatter diagram of the WC-M2 composite coatings. These images reveal that within the larger blocky crystals, numerous fine grains are embedded. Figure 6(a2,b2,c2,d2) clearly illustrates the presence of bands within the coatings, characterized by variously colored blocky crystals. The multicolored bands indicate different crystal orientations, suggesting that the blocky crystals form at specific angles [22]. Figure 6(a3,b3,c3,d3) display the phase distribution in the coatings. As indicated in Figure 6(a3,b3), in WC 20 and WC30, the hard phases are primarily MC and WC_x_. However, as depicted in Figure 6(c3,d3), as the WC content increases to 40 wt.% and 50 wt.%, the hard phases in the coating transition to being primarily Fe-W-C phases with higher hardness. With the rise in WC content, the hit rate of the EBSD analysis for the WC-M2 composite coating notably decreased, indicating that the coating contains a greater amount of precipitated hard phases and that these phases exhibit higher hardness [23].

**Figure 7:**
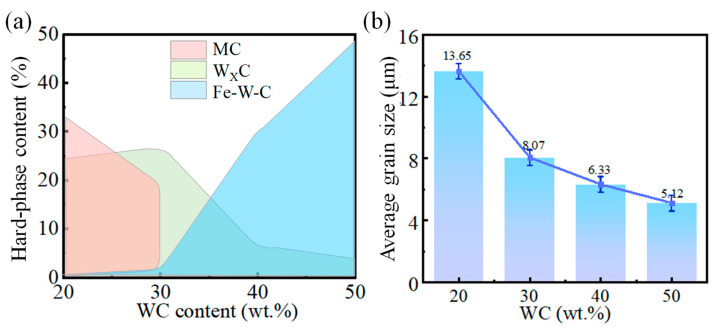
WC-M2 composite coating with different WC content; (**a**) hard phase content; (**b**) average grain size.

As depicted in Figure 7a, as the WC content varies from 20 wt.% to 30 wt.%, the hard phases in the coating are predominantly MC and W_x_C, which are carbides resulting from the precipitation of W and C elements from WC. At a WC content of 40 wt.%, Fe_3_W_3_C emerges in the coating, signifying the diffusion between W and C elements and the Fe matrix [24]. A further increase in WC content leads to some Fe_3_W_3_C transforming into the more stable Fe_6_W_6_C and W_X_C [14,25]. The existence of hard phases within the coating is responsible for its increased microhardness and wear resistance. As observed in Figure 7b, with rising WC content, coating grain size refines, albeit with a diminishing trend in fineness. Average grain sizes of the WC-M2 coatings are 13.65, 8.07, 6.33, and 5.12 μm, respectively. The rate of cooling is a direct determinant of grain size [26]. WC exhibits exceptional thermal conductivity [12]. With the increase in WC content, the increase in residual WC particles within the coating fosters the development of thermal conduction pathways [9], significantly enhancing heat transfer efficiency within the coating. This heightened thermal conductivity ensures that heat is conveyed to the substrate and dissipated, accelerating the cooling process of the coating [21]. Moreover, during laser irradiation, WC particles absorb some energy, moderating the temperature increase in the coating. During the solidification process, achieving sufficient constitutional undercooling prior to the solid–liquid interface is imperative for effectively minimizing the grain size in metal alloys [19]. High cooling rates significantly amplify the thermal gradients during the laser cladding process, fostering supercooling in the melt pool alloy, suppressing secondary dendrite arm growth, and facilitating grain refinement.

### 3.3. Microhardness

**Figure 8:**
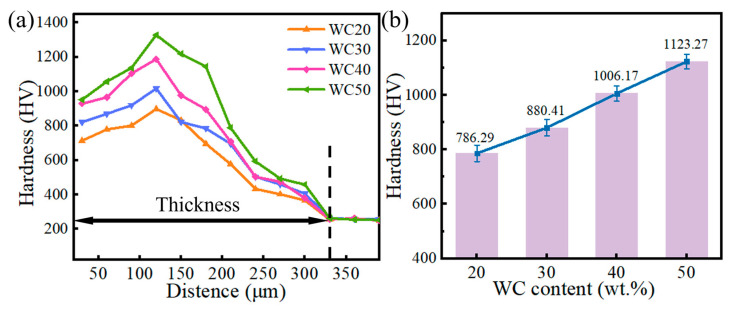
WC-M2 composite coating with different WC content. (**a**) Microhardness curve; (**b**) average hardness.

The microhardness variation depicted in Figure 8 highlights that the WC-M2 composite coating exhibits significantly higher hardness than the H13 substrate, with a gradual increase observed in correlation with the WC content. The primary factors are as follows: (1) with increasing WC content, the proportion of undecomposed WC particles in the coating augments, and the average particle size expands, thereby strengthening the coating through particle reinforcement; (2) the augmentation in C and W elements within the coating induces solid solution strengthening; (3) hard phases such as WC, W_2_C, and Fe_3_W_3_C contribute to precipitation and dispersion strengthening; (4) the grain refinement effect is pronounced, with an escalation in the area of phase interfaces, which impedes dislocation movement and elevates the microhardness of the coating [27,28].

### 3.4. Toughness

**Figure 9:**
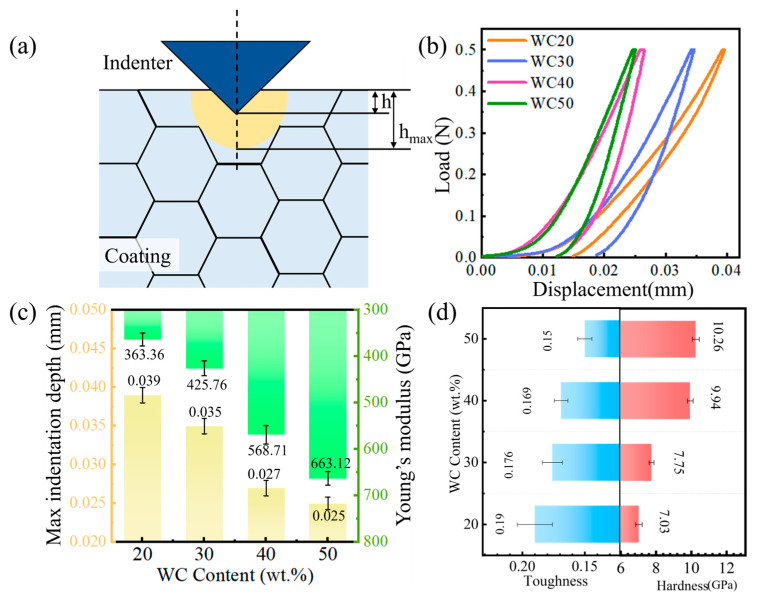
(**a**) Illustration of micro-indentation; WC-M2 composite coating: (**b**) L–D curve; (**c**) Young’s modulus and max indentation depth; (**d**) toughness and hardness.

The hardness and toughness of a coating are critical properties, yet they commonly display a trade-off relationship; enhancing hardness typically results in a reduction in toughness [8]. Micro-indentation can be used to characterize the toughness of the coating, as illustrated in Figure 9a. h_max_ denotes the maximum indentation depth, and upon the removal of the applied force, the material undergoes partial deformation, resulting in a reduction in the indentation depth, which is finally denoted by h [28,29]. Figure 9b illustrates the load–displacement curve for the WC-M2 coating, where it is observed that the gradient of the curve steepens progressively as the WC content is augmented. The Oliver-Pharr method was used to evaluate the Young modulus and hardness [26]:

(1) $$E^{*}=\frac{E}{{1-\nu }^{2}}$$

(2) $$T=\frac{H}{{\;E}^{*}}$$

where E* is the effective Young modulus, E and v are the Young modulus and Poisson ratio of the coating, H is the hardness of the coating, and T is the toughness of the coating.

The maximum indentation depth of the indenter and Young’s modulus are shown in Figure 9c, and the calculation results of toughness and hardness are shown in Figure 9d. The h_max_ of the coating decreases, indicating higher deformation resistance due to more WC particles, thus enhancing hardness, with increasing WC content. As depicted in Figure 2 and Figure 3, WC particles are dispersed throughout the coating. As the WC content increases from 40 wt.% to 50 wt.%, upon saturation of W and C in the melt pool, WC particles are significantly retained, leading to an increase in their concentration within the coating. These undecomposed WC contribute to the reinforcement of the coating, effectively bolstering its hardness. Notably, the calculated Young modulus values appear relatively high (360–670 GPa), which could be attributed to the high volume fraction of hard phases, such as WC_x_ and Fe_6_W_6_C, and the small grain sizes, as observed in the EBSD results in Figure 6. During the nano-indentation process, the indenter could penetrate to these hard phases, resulting in a higher measured hardness, and subsequently, a higher estimated Young’s modulus. Nevertheless, WC particles also increase the defects and internal stress of the coating, reducing the compactness and uniformity and diminishing its plastic deformation capacity, leading to a decrease in coating toughness.

### 3.5. Wear Resistance

**Figure 10:**
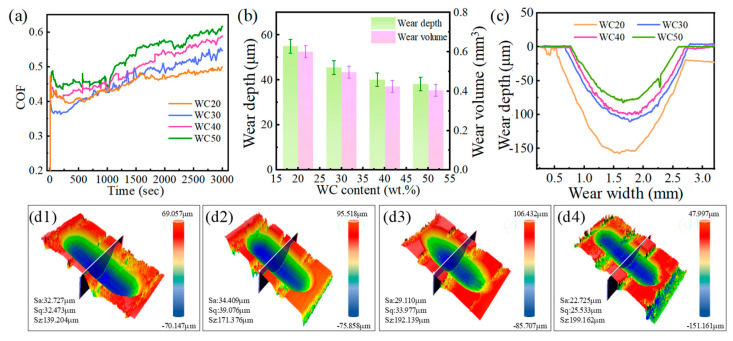
(**a**) Friction coefficient curve of the WC-M2 composite coating; (**b**) wear depth and wear volume; (**c**) the 2D wear morphology of the WC-M2 composite coating; (**d1**–**d4**) the 3D wear morphology of the WC-M2 composite coating: (**d1**) WC20; (**d2**) WC30; (**d3**) WC40; (**d4**) WC50.

As depicted in Figure 10a, the friction coefficient of the WC-M2 composite coating rises with increasing WC content. Respectively, the average friction coefficients for the WC-M2 coatings are 0.446, 0.461, 0.494, and 0.523. With the WC content increasing from 20 wt.% to 50 wt.%, Figure 3 reveals a notable increase in both the dimensions and quantity of WC particles within the coating, resulting in an elevation of the friction coefficient of the coating [30]. Figure 10(c,d1–d4) depict the 2D and 3D wear morphologies of the WC-M2 composite coating. In line with tribological principles [31] and fatigue theory [32,33], a higher hardness correlates with enhanced resistance to both adhesive and abrasive wear mechanisms, implying that the wear durability of the material is primarily determined by two key factors: its inherent hardness and its capacity to resist fracture [34]. As evidenced by Figure 8 and Figure 9, the hardness of the coating experiences a corresponding enhancement, and its toughness decreases with growing WC content. As shown in Figure 10b, the increase in WC content results in narrower and shallower wear tracks, leading to a reduction in wear volume. To evaluate the wear resistance of the coating, the specific wear rate is employed, which is expressed as

(3) $$\mathrm{Specific}\;\mathrm{wear}\;\mathrm{rate}=\frac{V}{\mathrm{FL}}$$

where V is the volume loss, F is the normal load, and L is the sliding distance.

A lower specific wear rate indicates a higher wear resistance of the material. As the WC content increases, the wear volume decreases, accompanied by a reduction in the specific wear rate, indicating that the incorporation of WC as a reinforcing phase significantly enhances the wear resistance. However, this improvement is observed to be tapering off, with the wear volume decreasing by 29.84% when the WC content increases from 20 wt.% to 30 wt.% and by only 9.81% when it increases from 40 wt.% to 50 wt.%. The coating prepared via laser cladding was approximately 300 μm thick. After wear testing at 500 °C for 1 h under a high contact stress, the wear depth was found to be less than 20% of the coating thickness, indicating its excellent combination of toughness and hardness.

**Figure 11:**
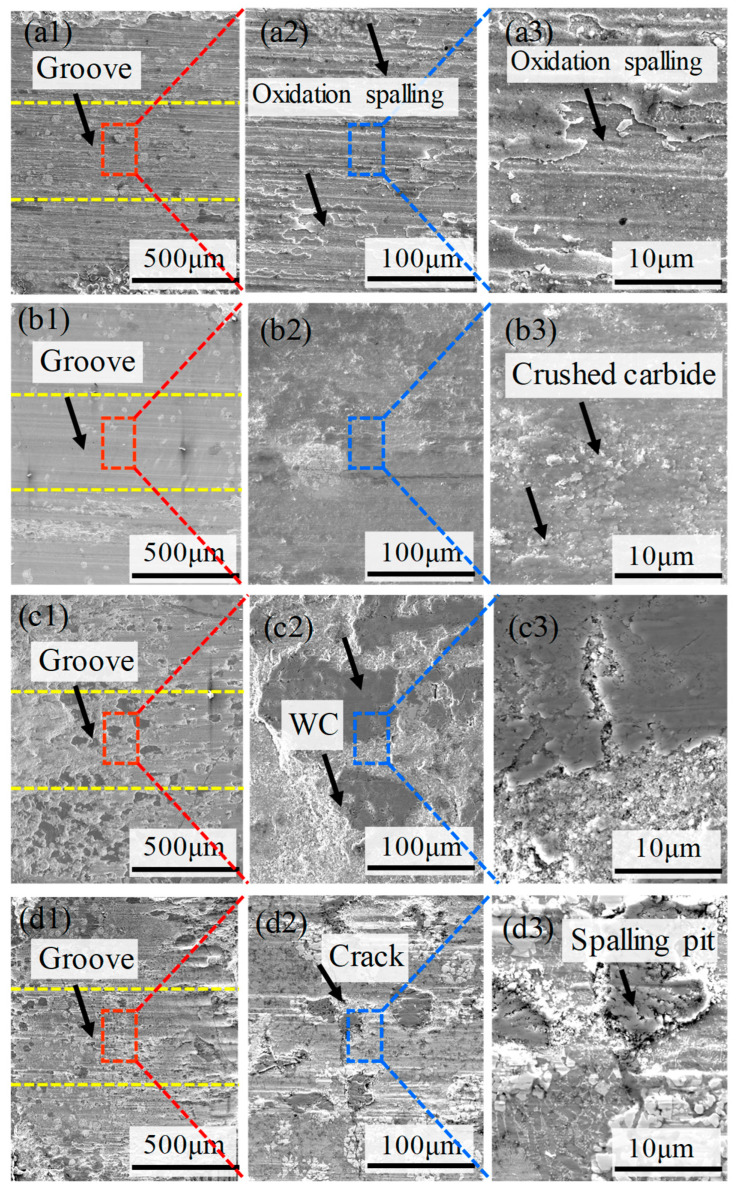
(**a1**–**a3**) Wear surface morphology of the WC-M2 composite coating: (**a1**–**a3**) WC20; (**b1**–**b3**) WC30; (**c1**–**c3**) WC40; (**d1**–**d3**) WC50.

Figure 11(a1–a3, b1–b3) illustrate that the surfaces of WC20 and WC30 show signs of plastic deformation and oxidative spalling. At this stage of the coating, the wear surfaces are marked by closely spaced grooves that are parallel to the direction of sliding, indicating abrasive wear characterized by micro-cutting. This is attributed to the low volume and the small size of WC particles in the coating when the WC content is from 20 wt.% to 30 wt.%. At this WC content, the microstructure of the coating demonstrates the first type of solidification, with fine eutectic carbides and an increased fraction of bonding metal M2. This results in the preferential wear of M2 during the initial wear phase. The high-hardness eutectic carbides function as a protective barrier, effectively isolating the bonding metal M2 from the abrasive particles, thereby augmenting the wear resistance of the coating [30,35]. However, in coatings with lower WC content, the structure of the eutectic carbides is relatively fine and dispersed, rendering them susceptible to fracturing under continuous loading. Such fractured carbide fragments are readily incorporated into the wear track, serving as abrasive elements [36].

The wear extent continuously decreases as the WC content rises. At 40 wt.% WC content, the volume concentration of WC particles significantly increases, as does the particle size. WC particles are distinguished by their high microhardness and inherent wear resistance, and they serve as a structural framework within the Fe-based coating [21,37]. For the increase in W content within the coating, its microstructure transitions to the second type of solidification, yielding coarser and denser eutectic carbides and, thus, offering robust support to the matrix. At this stage, the coating primarily undergoes abrasive wear. The WC particles continue to wear until they fracture, transforming into abrasive particles. This shift changes the wear mechanism from two-body to three-body abrasive wear between the friction pairs [38]. However, when the WC content reaches 50 wt.%, the volume of WC particles in the coating greatly increases, with larger blocks and a multitude of sub-grains interspersed, triggering the formation of cracks and the nucleation and spalling of pits, which collectively intensify the wear of the coating. In conclusion, the wear resistance markedly improves with increasing WC content up to 40 wt.%, but beyond this threshold, within the 40 to 50 wt.% range, the wear resistance remains relatively constant.

**Figure 12:**
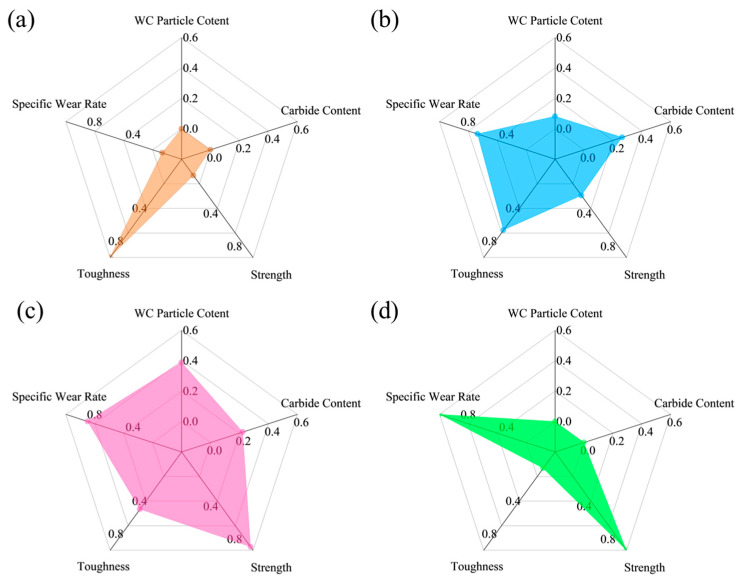
Radar diagrams of the overall performance of the WC-M2 composite coating: (**a**) WC20; (**b**) WC30; (**c**) WC40; (**d**) WC50.

To comprehensively evaluate the performance of the coating, a normalization process was applied to the data, encompassing WC particle content (macroscopic organization), carbide content (microscopic organization), strength, toughness, and specific wear rate. As shown in Figure 12, this transformation standardizes data of varying scales, facilitating comparative analysis and comprehensive evaluation. The strength and toughness of the coating are generally regarded as beneficial traits that increase with intensity, as they signify enhanced resistance to external forces and shock, ensuring the stability of the coating [18,23]. Conversely, a lower specific wear rate suggests the coating can sustain its functionality for an extended duration during use [39,40,41]. The WC particles and eutectic carbides in the coating, however, can influence its performance in multiple ways. As the volume fraction of WC particles increases, the strength and hardness of the coating commonly rise. Furthermore, excessive eutectic carbides can cause localized regions of the coating to become excessively hard, increasing the risk of cracking or peeling [26,42]. It also weakens the interfacial bonding strength, reducing the coating’s toughness and making it more susceptible to fracture under impact [25]. While high-hardness carbides enhance the wear resistance of the coating, their excess can lead to microcracks and defects that accelerate the wear process [9]. This suggests that the presence of WC particles and eutectic carbides does not necessarily correlate positively with improved performance. Consequently, as the volume concentration of WC particles and eutectic carbides is normalized, the value tends to converge to approximately 0.5, at which point the coating’s performance reaches its peak, striking a harmonious balance between hardness, wear resistance, toughness, and stability. Figure 11 reveals that the overall performance is at its zenith when the WC content is 40 wt.%.

## 4. Conclusions

In this study, four distinct WC-M2 composite coatings with WC contents of 20 wt.%, 30 wt.%, 40 wt.%, and 50 wt.% were prepared by laser cladding on H13 steel. The overall performance of the WC-M2 composite coatings was contrasted and analyzed. The primary findings are as follows:

(1) The microstructure of the coatings exhibited two distinctive solidification behaviors. When the WC content increases from 20 wt.% to 30 wt.%, the coating microstructure exhibits a fine network of eutectic carbides with hard phases of MC_X_ and WC_X._ With a further increase to 40–50 wt.%, the microstructure transforms to blocky, floral eutectics, where the hard phases change to Fe-W-C and W_X_C.

(2) The wear mechanism is influenced by distinct solidification behavior within the microstructure of the coating. At 20 wt.%–30 wt.% WC, the wear is dominated by plastic deformation and micro-cutting. At 40 wt.%, the wear mode transitions to abrasive wear, and at 50 wt.%, cracking and spalling become the primary wear modes.

(3) The coating achieved its optimal overall performance at 40 wt.% WC. Increasing WC content enhanced the hardness and wear resistance of the coating but compromised its stability and toughness.

## Figures and Tables

**Table 1:** Chemical composition of H13 and M2 (mass fraction wt.%).

Type	C	Cr	Si	Mn	Mo	Fe
M2	0.79	4.07	0.23	0.34	5.20	Balanced
H13	0.42	5.21	1.05	0.31	1.48	Balanced

**Table 2:** Laser cladding process experimental parameters.

Laser Cladding Process	
Scanning speed (mm/s)	5
*Z*-axis lift h (mm)	0.4
Carrier gas speed Q (L/min)	12
Laser power P (W)	2000
Powder feeder speed (rpm)	3
Laser spot diameter(mm)	2
Partical size range(µm)	40–45

**Table 3:** Wear process experimental parameters.

Wear Process	
Load F (N)	50
Wear length s (mm)	4
Frequency f (Hz)	12
Friction time t (min)	50
Friction temperature T (°C)	500

## Data Availability

The experimental data presented in this paper are the property of the research group and are subject to confidentiality agreements. As such, the data can only be made available upon authorization by the corresponding author and cannot be publicly disclosed.

## Abbreviations

The following abbreviations are used in this manuscript:

EH-LIHC	Electrothermal Heating-Laser Induced Hot Coating
EDS	Energy-Dispersive Spectrometer
EBSD	Electron Backscatter Diffraction
XRD	X-ray diffraction
SEM	Scanning Electron Microscopy
TEM	Transmission Electron Microscopy
HRTEM	High-Resolution Transmission Electron Microscopy
